# Anxiety and Depression Among People Under the Nationwide Partial Lockdown in Vietnam

**DOI:** 10.3389/fpubh.2020.589359

**Published:** 2020-10-29

**Authors:** Huong Thi Le, Andre Jun Xian Lai, Jiaqian Sun, Men Thi Hoang, Linh Gia Vu, Hai Quang Pham, Trang Ha Nguyen, Bach Xuan Tran, Carl A. Latkin, Xuan Thi Thanh Le, Thao Thanh Nguyen, Quan Thi Pham, Nhung Thi Kim Ta, Quynh Thi Nguyen, Roger C. M. Ho, Cyrus S. H. Ho

**Affiliations:** ^1^Institute for Preventive Medicine and Public Health, Hanoi Medical University, Hanoi, Vietnam; ^2^Department of Psychological Medicine, Yong Loo Lin School of Medicine, National University of Singapore, Singapore, Singapore; ^3^Institute for Global Health Innovations, Duy Tan University, Da Nang, Vietnam; ^4^Faculty of Medicine, Duy Tan University, Da Nang, Vietnam; ^5^Center of Excellence in Evidence-based Medicine, Nguyen Tat Thanh University, Ho Chi Minh City, Vietnam; ^6^Bloomberg School of Public Health, John Hopkins University, Baltimore, MD, United States; ^7^Institute for Health Innovation and Technology (iHealthtech), National University of Singapore, Singapore, Singapore; ^8^Department of Psychological Medicine, National University Hospital, Singapore, Singapore

**Keywords:** COVID-19, anxiety, depression, lockdown, Vietnam

## Abstract

This study aimed to evaluate the psychological effects of the partial lockdown on the people in Vietnam during the COVID-19 pandemic. An online questionnaire regarding attitudes toward COVID-19 along with psychological parameters, including the Impact of Event Scale-Revised (IES-R) Depression, Anxiety, Stress Scale-21 (DASS-21) was conducted. From a total of 1,382 questionnaires, the respondents reported low prevalence of depression (4.9%), anxiety (7.0%), and stress (3.4%). The mean DASS-21 scores recorded were also markedly lower compared to similar studies conducted in China, Italy, and Iran. Respondents who reported severe PTSD had significantly higher depression, anxiety, and stress levels. Factors that were associated with an increased level of depression, stress, and anxiety were being single, separated, or widowed, a higher education level, a larger family size, loss of jobs and being in contact with potential COVID-19 patients. Contrary to expectations, the level of depression, stress, and anxiety observed has been low. Our findings can aid in future research on the impact of a partial lockdown and guide mental health professionals in Vietnam and other countries in the preparation of better care for populations under such circumstances.

## Introduction

The 2019 outbreak of Coronavirus Disease (COVID-19) was first documented in Wuhan, China in early December 2019 and resulted in over 16 million infected and over 600,000 deaths worldwide to date. The World Health Organisation (WHO) has since declared it a pandemic. Following the outbreak, the Vietnam government tightened border control and implemented social distancing measures. With the increasing numbers of cases locally and worldwide, Vietnam implemented a partial lockdown on April 1, 2020 where residents must stay at home and can only go outside for “essential needs.” “Essential needs” referred to buying food, medicine, emergency circumstances, going to work at factories and businesses that do not close or suspend their operations. The nationwide lockdown lasted 2 weeks, with an extension for 12 of the major cities. Jobs were affected, with many workers left without a job or were furloughed as the economy ground to a halt. With prolonged quarantines, restrictions on public life and worries of job security, there might be rising levels of psychiatric issues such as anxiety, stress, and depression amongst the population (1, 2).

The emphasis of research during this pandemic has been on cures and vaccines for COVID-19, and research on the psychosomatic impact and mental well-being of COVID-19 have mostly been focusing on the outbreak in China, from the general population, to psychiatric patients, healthcare workers and those returning to work after a lockdown (3–7). Currently, to our knowledge, there are no studies on the psychological impact of the partial lockdown in Vietnam. This pa*per seeks* to address the psychiatric problems such as anxiety, depression, and stress amongst the Vietnamese population, as well as explore the factors associated with mental health conditions during the nationwide partial lockdown that was implemented on the April 1, 2020.

## Methods

### Study Design

A cross-sectional study was conducted using a web-based questionnaire. This is a sub-analysis of a project on COVID-19 pandemic. The study was carried out within 1 week of national lockdown, enacted on April 1, 2020 due to the COVID-19 pandemic in Vietnam. The inclusion criteria were: (1) Giving online informed consent for the survey, (2) Having the ability to access the questionnaire online, and (3) Having the ability to understand and respond to the questionnaire.

The snowball sampling technique was used to recruit the respondents. Firstly, staff, and medical students at Hanoi Medical University and Hanoi Medical University Hospital were invited to enroll in the study through social media. In addition to their participation, the respondents were encouraged to invite their contacts to participate in the survey.

A total of 1,382 respondents (including hospital and medical university staff, medical students, teachers, officers, and other community members) throughout all 63 provinces of Vietnam were sampled within 1 week.

### Variable

The survey included questions on (1) social-economics characteristics, (2) respondents' comorbidities, (3) whether they underwent COVID-19 testing and quarantine, (4) Depression, Anxiety and Stress Scale (DASS-21), and (5) Impact of Event Scale—Revised (IES-R).

#### Social— Demographic

Social demographic characteristics including gender, region, age, marital status, occupation, number of children, and educational level.

#### Psychosocial Status

The DASS-21 is a subjective measure in which respondents rate the frequency and severity of experiencing negative emotions over the last week based on 42 questions. The ratings of the DASS scale is as follows from 1—Did not apply to me at all to 4—applied to me most of the time. There are three subscale domains divided from the scale: depression, anxiety, and stress. The calculated scores can then be used to stratify patients into varying severities of depression, anxiety and stress in which the higher the score, the greater the severity of symptoms.

#### Impact of COVID 19

The Impact of Event Scale—Revised (IES-R) was used to assess subjective distress caused by traumatic events. This scale has 22-item self-report with a 5-point scale ranging from 0 “not at all” to 4 “extremely” for each item. The IES-R yields a total score ranging from 0 to 88 and is divided into four domains: “Normal,” “Clinical concern,” “Post-traumatic stress disorder diagnosis,” and “Severe post-traumatic stress disorder.”

Respondents were asked about the Impact of COVID 19 on their income and occupation status.

Additionally, we collected data on respondents' comorbidities, whether they underwent COVID testing and if they were subjected to a 14-day quarantine.

### Data Analysis

STATA 15.0 software was used to analyze the data. Descriptive statistics were used to examine characteristic data, including frequency, percent, mean, and standard deviation. Inferential statistics were applied to perform the comparison among two subject groups by the Fisher-exact test or Chi-squared test for qualitative variables and by *T*-test or Mann Whitney test for quantitative variables. A general multivariable regression model was used to identify factors associated with depression, anxiety, stress. To achieve reduced models, stepwise forward selection strategies were utilized with a log-likelihood ratio test at a *p*-value of 0.2. Statistical significance was defined at a *p*-value of < 0.05.

### Ethical Consideration

The Review Committee at the Institute for Preventive Medicine and Public Health, Hanoi Medical University approved the research on March 28, 2020. The purpose of research and informed consent were available on the online web-based survey for participants to examine prior to deciding on participating. Participation was voluntary and anonymity was assured. Respondents could decline to participate or withdraw from the online survey at any time.

## Results

### Tables

Table 1 shows the information about respondents' socio-economics characteristics. One thousand three hundred and eighty two valid questionnaires were analyzed. The mean age of the respondents was 36.4 (SD = 9.7). The majority of the respondents were female (62.0%, *n* = 857). A total of 76.7% were married, and 23.3% were single, divorced, or widowed. Most respondents lived in a household of 3–5 family members (73.2%), and the mean number of children in the family was 1.3 (SD = 0.9). Majority of the respondents were from Northern Vietnam (47.1%) and belonged to the 25–34 age group (40.2%). Forty-seven percent of respondents reported that their income decreased due to COVID 19. In terms of education levels, most of the respondents had the highest attained education level of undergraduate (79.9%). In terms of occupation, the respondents are mainly healthcare workers (69.7%) and workers permanently employed by their companies (64.7%).

**Table 1:** Socioeconomics characteristics of participants.

	**Impact of COVID 19 on income**				**Total**		***p*-value**
	**Decreased**		**No change**				
	***n***	**%**	***n***	**%**	***n***	**%**	
**Total**	650	47.0	732	53.0	1,382	100.0	
**Region**							
Northern	313	48.2	338	46.2	651	47.1	0.17
Central	220	33.8	282	38.5	502	36.3	
South	112	17.2	110	15.0	222	16.1	
Foreign	5	0.8	2	0.3	7	0.5	
**Gender**							
Male	226	34.8	299	40.8	525	38.0	0.02
Female	424	65.2	433	59.2	857	62.0	
**Age group**							
Under 25	65	10.0	48	6.6	113	8.2	0.01
25–34	279	42.9	277	37.8	556	40.2	
35–44	176	27.1	244	33.3	420	30.4	
Above 44	130	20.0	163	22.3	293	21.2	
**Marital status**							
Single/ Separated/ Widowed	165	25.4	157	21.4	322	23.3	<0.01
Married	485	74.6	575	78.6	1,060	76.7	
**Family size**							
1 - 2 people	99	15.2	115	15.7	214	15.5	0.53
3 - 5 people	471	72.5	541	73.9	1,012	73.2	
More than 5 people	80	12.3	76	10.4	156	11.3	
**Education level**							
High school and below	49	7.5	51	7.0	100	7.2	<0.01
Undergraduate	496	76.3	608	83.1	1,104	79.9	
Postgraduate	105	16.2	73	10.0	178	12.9	
**Occupation**							
Health workers	401	61.7	562	76.8	963	69.7	<0.01
Professional educators	39	6.0	30	4.1	69	5.0	
White collar workers	84	12.9	60	8.2	144	10.4	
Students	55	8.5	37	5.1	92	6.7	
Others	71	10.9	43	5.9	114	8.2	
**Occupation status**							
Functionary	361	55.5	533	72.8	894	64.7	<0.01
Unlimited term fulltime contract	107	16.5	82	11.2	189	13.7	
Limited term fulltime contract	75	11.5	54	7.4	129	9.3	
Farmers/ Students/ Homemakers/ Unemployed/ Retired	74	11.4	48	6.6	122	8.8	
Others	33	5.1	15	2.0	48	3.5	
	**Mean**	**SD**	**Mean**	**SD**	**Mean**	**SD**	***p*****-value**
**Age**	35.6	9.4	37.0	9.9	36.4	9.7	0.01
**Number of children**	1.3	1.0	1.4	0.9	1.3	0.9	0.51

Table 2 shows the impact of COVID-19 reported by the respondents. About 2.7% underwent COVID-19 testing, 3.7% had been isolated in the last 14 days, and 98.2% had health insurance. 56.4% of the respondents reported that COVID-19 had affected their occupation status, while 47.0% reported decreased income due to the outbreak. In terms of history of contact to COVID-19, 13.5% of respondents had contact with suspected COVID-19 infected people, 7.2% had contact with people who were directly exposed to COVID-19 patients, and 4.0% had contact with fomites possibly containing COVID-19.

**Table 2:** Impact of COVID-19 on participant life.

	**Total**	
	***n***	**%**
**Had outpatient examination in last 14 days**	74	5.4
**Had COVID-19 test in last 14 days**	37	2.7
**Been isolated in last 14 days**	51	3.7
**Have health insurance**	1,356	98.2
**COVID 19 impact on occupation status**		
No effect	602	43.6
Fired	39	2.8
Reduced working hours/shift	217	15.7
Have to work overtime	524	37.9
**Amount of income effected by COVID-19**		
Increased 80–100%	1	0.1
Increased 60–80%	0	0.0
Increased 40–60%	1	0.1
Increased 20–40%	3	0.2
Increased <20%	3	0.2
No change	724	52.4
Decreased <20%	202	14.6
Decreased 20–40%	189	13.7
Decreased 40–60%	168	12.2
Decreased 60–80%	56	4.1
Decreased 80–100%	35	2.5
**History of contact to COVID-19**		
Close contact with COVID-19 infected person	25	1.8
Contact with people who have directly exposed to COVID-19 patient	100	7.2
Contact with suspected COVID-19 infected people	186	13.5
Contact with objects which possibility contain the COVID-19 virus	55	4.0
Never had contact with COVID-19 infected person	1,099	79.5

Table 3 shows the mental well-being of respondents during COVID-19. Based on DASS 21 scale scoring, 4.9% of respondents were classified as having moderate to extremely severe levels of depression, 7.0% of respondents had moderate to extremely severe levels of anxiety, and 3.4% of respondents scored moderate to extremely severe levels of stress. Mean scores for depression, anxiety, and stress were 2.7 (SD = 6.5), 2.4 (SD = 6.3), and 4.7 (SD = 7.0), respectively. Most respondents had normal levels of stress caused by COVID 19 (74.3%) with the IES-R score was 16.3 ± 13.3. There were no significant differences observed in the depression, anxiety, and stress scores and IES-R score based on the impact of COVID-19 on income among the respondents.

**Table 3:** Mental well-being of respondents during COVID-19.

	**Impact of COVID 19 on income**				**Total**		***p*-value**
	**Decreased**		**No change**				
	***n***	**%**	***n***	**%**	***n***	**%**	
**Depression**							
Normal	576	88.6	688	94.0	1,264	91.5	<0.01
Mild	36	5.5	13	1.8	49	3.5	
Moderate	21	3.2	14	1.9	35	2.5	
Severe	2	0.3	1	0.1	3	0.2	
Extremely severe	15	2.3	16	2.2	31	2.2	
**Anxiety**							
Normal	584	89.8	678	92.6	1,262	91.3	0.23
Mild	14	2.2	10	1.4	24	1.7	
Moderate	34	5.2	23	3.1	57	4.1	
Severe	3	0.5	2	0.3	5	0.4	
Extremely severe	15	2.3	19	2.6	34	2.5	
**Stress**							
Normal	607	93.4	701	95.8	1,308	94.6	0.03
Mild	19	2.9	8	1.1	27	2.0	
Moderate	8	1.2	5	0.7	13	0.9	
Severe	6	0.9	2	0.3	8	0.6	
Extremely severe	10	1.5	16	2.2	26	1.9	
**IES-R score interpretation**							
Not concerned at all	449	69.1	578	79.0	1,027	74.3	<0.01
Rarely concerned	100	15.4	93	12.7	193	14.0	
Concerned	46	7.1	22	3.0	68	4.9	
Extremely concerned	55	8.5	39	5.3	94	6.8	
	**Mean**	**SD**	**Mean**	**SD**	**Mean**	**SD**	***p*****-value**
**DASS 21 score**							
Depression (band score: 0–42)	3.2	7.6	2.4	5.8	2.7	6.5	0.27
Anxiety (band score: 0–42)	2.8	7.4	2.1	5.5	2.4	6.3	0.92
Stress (band score: 0–42)	4.9	7.7	4.6	6.5	4.7	7.0	0.78
**IES-R score** (band score: 0–88)	16.5	13.4	16.3	13.3	16.3	13.3	0.76

Table 4 shows the factor associated with the depression, anxiety, and stress related to COVID-19. Married respondents had lower depression, anxiety, and stress levels than those that were single, separated, or widowed. Participants who are postgraduates had significantly higher stress levels than those with education level of high school and below (*p* < 0.05). A larger family size of more than 5 people was also linked to higher levels of depression, anxiety, and stress (*p* < 0.1) than those with family size of 1–2 people. Respondents who were fired were more likely to be depressed (*p* < 0.05). Compared to respondents reporting normal levels on the IES-R scale, those who reported severe PTSD had significantly higher depression, anxiety, and stress levels with *p*-value were < 0.01. Respondents who had IES-R score in the partial PTSD (*p* < 0.01) and diagnosis of PTSD range (*p* < 0.01) also reported significantly higher levels of depression, anxiety and stress levels than those with normal scores. Coming into contact with suspected COVID-19 patients made participants more stressed than those who had no contact. There was also a possible association between people coming into contact with fomites possibly containing the COVID-19 virus and higher anxiety levels (*p* < 0.05).

**Table 4:** Associated factors with depression, anxiety and stress related to COVID 19 among Vietnamese.

	**Depression**		**Anxiety**		**Stress**	
	**Coef**.	**95% CI**	**Coef**.	**95% CI**	**Coef**.	**95% CI**
**Region** (vs. Northern)						
Central	−1.42	−3.29; 0.45				
**Gender** (vs. Male)						
Female	−1.53^*^	−3.33; 0.27				
**Marital status** (vs. Single/Separated/Widowed)						
Married	−3.69^***^	−5.74; −1.65	−2.43^**^	−4.48; −0.39	−1.80^***^	−3.10; −0.50
**Family size** (vs. 1 - 2 people)						
More than 5 people	2.69^*^	−0.01; 5.39			1.57^*^	−0.13; 3.27
**Education level** (vs. High school and below)						
Undergraduate	−2.09^*^	−4.22; 0.04				
Postgraduate					1.95^**^	0.36; 3.55
**Occupation** (vs. Health workers)						
Others	1.96	−1.10; 5.03			1.44	−0.54; 3.41
**COVID 19 impact on occupation status** (vs. No effect)						
Fired	5.27^**^	0.32; 10.22			3.22^*^	−0.16; 6.60
Reduced working hours/shift	1.83	−0.72; 4.38			1.42^*^	−0.20; 3.04
Have to work overtime	0.34	−1.69; 2.38			1.17^*^	−0.10; 2.43
**Amount of income decreased due to COVID-19**	0.18	−0.47; 0.83	0.31	−0.31; 0.93	0.25	−0.15; 0.66
**IES-R score interpretation** (vs. Normal)						
Partial PTSD	4.36^***^	1.89; 6.83	3.31^***^	0.81; 5.82	3.68^***^	2.13; 5.23
Diagnosis of PTSD	8.47^***^	4.79; 12.15	6.92^***^	3.13; 10.70	8.06^***^	5.67; 10.46
Severe PTSD	10.88^***^	7.66; 14.09	12.50^***^	9.35; 15.65	9.15^***^	7.06; 11.25
**History of contact to COVID-19** (Yes vs. No)						
Contact with suspected COVID-19 infected people					2.07^**^	0.45; 3.69
Contact with objects which possibility contain the COVID-19 virus			5.91^***^	1.83; 9.98		

*** *p < 0.01*,

** *p < 0.05*,

* *p < 0.1*.

## Discussion

As this study was conducted within the first week of the partial lockdown in Vietnam, it was able to capture the burden of COVID-19 on the Vietnamese population at the pinnacle of the pandemic.

### Key Findings

There is generally a low prevalence of reported depression, anxiety, and stress symptoms based on the surveys. The mean DASS-21 depression, anxiety, and stress scores were lower than those previously reported in other countries, such as China, Iran, and Italy (8–11) Confidence in doctor's ability to diagnose or recognize, greater likelihood to survive COVID-19, and satisfaction with health information were associated with lower DASS depression, anxiety, stress, and IES-R scores (8). This finding explains our study's results with the Vietnam government's successful attempt at keeping infections low and death rate at zero, with early intervention and intensive control measures to curb the spread of the virus (12). Further surveys can be done to evaluate for such associations in this study population.

Our reports suggested that respondents who had an education level of postgraduate have significantly higher stress levels than those with an education level of high school and below (*p* < 0.05). One postulation that could explain this phenomenon could be that those with a higher education attainment have invested significantly more resources and as such may possibly have a higher expectation for their future employment. Additionally, higher education often comes with greater financial stress upon students because of steeper tuition fees. This effect of higher education and financial stress was explored in the UK, which showed that students with greater financial stress were associated with worse mental health outcomes (13). With COVID-19 causing many countries to close their borders, economies contracting as a result of reduced trade and companies forced to reduce the number of employees, the prospects within the job market remain bleak in terms of number of available spots and the impact on their potential salary. Higher expectations and financial stress of those with higher educational attainment coupled with the massive impact of COVID-19 on jobs could have contributed to them having greater levels of stress (14).

Our results suggest that respondents who were fired due to COVID-19 reported higher levels of stress, anxiety, and depression. When faced with job losses, individuals experience stress related conditions and even physical symptoms (15). Moreover, retrenched individuals are more likely to need to seek employment, which can further worsen the impact on their mental health (16). This further emphasizes the need for the government to step up efforts to ensure that families can receive reassurances from their companies about their job stability or future job opportunities. Secondly, the government should implement policies to enhance financial support for lower-income families and develop programs to enhance the support for the unemployed during such trying times.

Our results also suggest the respondents were more likely to experience stress after contact with a suspected case of COVID-19. Besides the fear of severe complications of COVID-19, it is likely that respondents are worried that they might have to be isolated after contracting the disease. Isolation could lead to loneliness and even lead to suicidal ideations (17, 18). Another postulation would be that respondents were more stressed because they were afraid that they would become asymptomatic carriers and propagate the transmission of the disease to others who may belong to a more vulnerable group (19).

While this study showed a correlation between the IES-R score and the prevalence of depression, anxiety, and stress, the likelihood of moderate to severe mental health burden measured by IES-R was greater than that of DASS-21. The main distinction between IES-R and DASS-21 is that IES-R measures the mental health burden after an event, which is COVID-19 in this case (20).

A larger family size is associated with higher levels of depression, anxiety, and stress (21). This association was also previously established in a South Korean study, which showed that a larger household size was associated with greater stress amongst family members (22). This may be due to increased levels of concern with other family members contracting the disease. Previous studies on the psychological impact of COVID-19 where about 75.2% reported they were worried or somewhat worried about other family members getting the disease (20). With larger families, there is greater financial stress on the breadwinners. It is important for mental health professionals to explore their concerns and focus on promoting positive coping styles, which were shown to be a protective factor against depression and anxiety during the COVID-19 outbreak (23, 24).

### Strengths and Limitations

This study has several strengths. One of the strengths is that our study is the first to evaluate the effect of the partial lockdown on the mental health of the Vietnamese population at the peak of the COVID-19 pandemic. As previous studies mainly focus on populations in China, very little is known about the effects of COVID-19 on countries that are further away from the origin and the possible effects it could have on their populations. Another strength of our paper is that it can serve as a guide for the Vietnamese policymakers and healthcare organizations to place further emphasis on the mental well-being of the population as one of the key indicators of the effectiveness of the partial lockdown.

This study has several limitations. Due to the COVID-19 outbreak and social distancing measures, the snowball sampling strategy was used to recruit respondents. This method may not be representative of the population. Sampling bias may also be presented as respondents tend to share the survey with their close friends who may share similar traits. Our study established that there are many factors associated with increased levels of stress, anxiety, and depression amongst respondents during the partial lockdown. While we assessed for associations between more common causative factors such as job losses, health and isolation, future studies can explore the reasons behind these associations which can help provide greater clarity and allow for more targeted interventions.

## Conclusion

Our paper provides information on factors affecting depression, anxiety, and stress during a nationwide lockdown. This study contributes to the existing literature to explore the acute effects of a lockdown on the mental health of the population. While long term psychological effects of the lockdown may yet to manifest, these early signs of anxiety, stress, and depression amongst the population can serve as a marker to predict the long term psychological impact. Furthermore, it raises the awareness on the topic of mental health during the COVID 19 pandemic, which will hopefully be addressed by organizations and governmental policies in the future to guide a greater holistic pandemic response. These results can guide policy making in future pandemics to take into account mental health issues faced by the public. This can improve psychological well-being of the population and create a more resilient country to get through such taxing times.

## Data Availability Statement

The raw data supporting the conclusions of this article will be made available by the authors, without undue reservation.

## Ethics Statement

The studies involving human participants were reviewed and approved by The Review Committee at the Institute for Preventive Medicine and Public Health, Hanoi Medical University. The patients/participants provided their written informed consent to participate in this study.

## Author Contributions

BT: conceptualization, methodology, supervision, writing—original draft, writing—review and editing. AL and JS: writing—original draft, writing—review and editing. HP: conceptualization, data curation, formal analysis, writing—review and editing. LV: data curation, formal analysis, writing—review and editing. MH: conceptualization, project administration, writing—review and editing. THN: writing—review and editing. HL: conceptualization, supervision, writing—review and editing. CL, CH, and RH: supervision, writing—review and editing. XL: conceptualization, methodology, supervision, writing—review and editing. TTN: data curation, project administration, writing—review and editing. QP, NT, and QN: project administration, writing—review and editing. All authors contributed to the article and approved the submitted version.

## Conflict of Interest

The authors declare that the research was conducted in the absence of any commercial or financial relationships that could be construed as a potential conflict of interest.
