# Corrigendum: Anxiety and Depression Among People Under the Nationwide Partial Lockdown in Vietnam

**DOI:** 10.3389/fpubh.2021.692085

**Published:** 2021-05-24

**Authors:** Huong Thi Le, Andre Jun Xian Lai, Jiaqian Sun, Men Thi Hoang, Linh Gia Vu, Hai Quang Pham, Trang Ha Nguyen, Bach Xuan Tran, Carl A. Latkin, Xuan Thi Thanh Le, Thao Thanh Nguyen, Quan Thi Pham, Nhung Thi Kim Ta, Quynh Thi Nguyen, Roger C. M. Ho, Cyrus S. H. Ho

**Affiliations:** ^1^Institute for Preventive Medicine and Public Health, Hanoi Medical University, Hanoi, Vietnam; ^2^Department of Psychological Medicine, Yong Loo Lin School of Medicine, National University of Singapore, Singapore, Singapore; ^3^Institute for Global Health Innovations, Duy Tan University, Da Nang, Vietnam; ^4^Faculty of Medicine, Duy Tan University, Da Nang, Vietnam; ^5^Center of Excellence in Evidence-based Medicine, Nguyen Tat Thanh University, Ho Chi Minh City, Vietnam; ^6^Bloomberg School of Public Health, John Hopkins University, Baltimore, MD, United States; ^7^Institute for Health Innovation and Technology (iHealthtech), National University of Singapore, Singapore, Singapore; ^8^Department of Psychological Medicine, National University Hospital, Singapore, Singapore

**Keywords:** COVID-19, anxiety, depression, lockdown, Vietnam

In the original article, there was a mistake in Table 1 as published. The corresponding author uploaded an incorrect table that did not match the relevant text in the result section. The corrected Table 1 appears below.

**Table 1:** Socioeconomics characteristics of participants.

	**Impact of COVID 19 on income**				**Total**		***p*-value**
	**Decreased**		**No change**				
	***n***	**%**	***n***	**%**	***n***	**%**	
**Total**	650	47.0	732	53.0	1,382	100.0	
**Region**							
Northern	313	48.2	338	46.2	651	47.1	0.17
Central	220	33.8	282	38.5	502	36.3	
South	112	17.2	110	15.0	222	16.1	
Foreign	5	0.8	2	0.3	7	0.5	
**Gender**							
Male	226	34.8	299	40.8	525	38.0	0.02
Female	424	65.2	433	59.2	857	62.0	
**Age group**							
Under 25	65	10.0	48	6.6	113	8.2	0.01
25–34	279	42.9	277	37.8	556	40.2	
35–44	176	27.1	244	33.3	420	30.4	
Above 44	130	20.0	163	22.3	293	21.2	
**Marital status**							
Single/ Separated/ Widowed	165	25.4	157	21.4	322	23.3	<0.01
Married	485	74.6	575	78.6	1,060	76.7	
**Family size**							
1 - 2 people	99	15.2	115	15.7	214	15.5	0.53
3 - 5 people	471	72.5	541	73.9	1,012	73.2	
More than 5 people	80	12.3	76	10.4	156	11.3	
**Education level**							
High school and below	49	7.5	51	7.0	100	7.2	<0.01
Undergraduate	496	76.3	608	83.1	1,104	79.9	
Postgraduate	105	16.2	73	10.0	178	12.9	
**Occupation**							
Health workers	401	61.7	562	76.8	963	69.7	<0.01
Professional educators	39	6.0	30	4.1	69	5.0	
White collar workers	84	12.9	60	8.2	144	10.4	
Students	55	8.5	37	5.1	92	6.7	
Others	71	10.9	43	5.9	114	8.2	
**Occupation status**							
Functionary	361	55.5	533	72.8	894	64.7	<0.01
Unlimited term fulltime contract	107	16.5	82	11.2	189	13.7	
Limited term fulltime contract	75	11.5	54	7.4	129	9.3	
Farmers/ Students/ Homemakers/ Unemployed/ Retired	74	11.4	48	6.6	122	8.8	
Others	33	5.1	15	2.0	48	3.5	
	**Mean**	**SD**	**Mean**	**SD**	**Mean**	**SD**	***p*****-value**
**Age**	35.6	9.4	37.0	9.9	36.4	9.7	0.01
**Number of children**	1.3	1.0	1.4	0.9	1.3	0.9	0.51

In the original article, there was a mistake in Table 2 as published. The corresponding author uploaded an incorrect table that did not match the relevant text in the result section. The corrected Table 2 appears below.

**Table 2:** Impact of COVID-19 on participant life.

	**Total**	
	***n***	**%**
**Had outpatient examination in last 14 days**	74	5.4
**Had COVID-19 test in last 14 days**	37	2.7
**Been isolated in last 14 days**	51	3.7
**Have health insurance**	1,356	98.2
**COVID 19 impact on occupation status**		
No effect	602	43.6
Fired	39	2.8
Reduced working hours/shift	217	15.7
Have to work overtime	524	37.9
**Amount of income effected by COVID-19**		
Increased 80–100%	1	0.1
Increased 60–80%	0	0.0
Increased 40–60%	1	0.1
Increased 20–40%	3	0.2
Increased <20%	3	0.2
No change	724	52.4
Decreased <20%	202	14.6
Decreased 20–40%	189	13.7
Decreased 40–60%	168	12.2
Decreased 60–80%	56	4.1
Decreased 80–100%	35	2.5
**History of contact to COVID-19**		
Close contact with COVID-19 infected person	25	1.8
Contact with people who have directly exposed to COVID-19 patient	100	7.2
Contact with suspected COVID-19 infected people	186	13.5
Contact with objects which possibility contain the COVID-19 virus	55	4.0
Never had contact with COVID-19 infected person	1,099	79.5

In the original article, there was a mistake in Table 3 as published. The corresponding author uploaded an incorrect table that did not match the relevant text in the result section. The corrected Table 3 appears below.

**Table 3:** Mental well-being of respondents during COVID-19.

	**Impact of COVID 19 on income**				**Total**		***p*-value**
	**Decreased**		**No change**				
	***n***	**%**	***n***	**%**	***n***	**%**	
**Depression**							
Normal	576	88.6	688	94.0	1,264	91.5	<0.01
Mild	36	5.5	13	1.8	49	3.5	
Moderate	21	3.2	14	1.9	35	2.5	
Severe	2	0.3	1	0.1	3	0.2	
Extremely severe	15	2.3	16	2.2	31	2.2	
**Anxiety**							
Normal	584	89.8	678	92.6	1,262	91.3	0.23
Mild	14	2.2	10	1.4	24	1.7	
Moderate	34	5.2	23	3.1	57	4.1	
Severe	3	0.5	2	0.3	5	0.4	
Extremely severe	15	2.3	19	2.6	34	2.5	
**Stress**							
Normal	607	93.4	701	95.8	1,308	94.6	0.03
Mild	19	2.9	8	1.1	27	2.0	
Moderate	8	1.2	5	0.7	13	0.9	
Severe	6	0.9	2	0.3	8	0.6	
Extremely severe	10	1.5	16	2.2	26	1.9	
**IES-R score interpretation**							
Not concerned at all	449	69.1	578	79.0	1,027	74.3	<0.01
Rarely concerned	100	15.4	93	12.7	193	14.0	
Concerned	46	7.1	22	3.0	68	4.9	
Extremely concerned	55	8.5	39	5.3	94	6.8	
	**Mean**	**SD**	**Mean**	**SD**	**Mean**	**SD**	***p*****-value**
**DASS 21 score**							
Depression (band score: 0–42)	3.2	7.6	2.4	5.8	2.7	6.5	0.27
Anxiety (band score: 0–42)	2.8	7.4	2.1	5.5	2.4	6.3	0.92
Stress (band score: 0–42)	4.9	7.7	4.6	6.5	4.7	7.0	0.78
**IES-R score** (band score: 0–88)	16.5	13.4	16.3	13.3	16.3	13.3	0.76

In the original article, there was a mistake in Table 4 as published. The corresponding author uploaded an incorrect table that did not match the relevant text in the result section. The corrected Table 4 appears below.

**Table 4:** Associated factors with depression, anxiety and stress related to COVID 19 among Vietnamese.

	**Depression**		**Anxiety**		**Stress**	
	**Coef**.	**95% CI**	**Coef**.	**95% CI**	**Coef**.	**95% CI**
**Region** (vs. Northern)						
Central	−1.42	−3.29; 0.45				
**Gender** (vs. Male)						
Female	−1.53^*^	−3.33; 0.27				
**Marital status** (vs. Single/Separated/Widowed)						
Married	−3.69^***^	−5.74; −1.65	−2.43^**^	−4.48; −0.39	−1.80^***^	−3.10; −0.50
**Family size** (vs. 1 - 2 people)						
More than 5 people	2.69^*^	−0.01; 5.39			1.57^*^	−0.13; 3.27
**Education level** (vs. High school and below)						
Undergraduate	−2.09^*^	−4.22; 0.04				
Postgraduate					1.95^**^	0.36; 3.55
**Occupation** (vs. Health workers)						
Others	1.96	−1.10; 5.03			1.44	−0.54; 3.41
**COVID 19 impact on occupation status** (vs. No effect)						
Fired	5.27^**^	0.32; 10.22			3.22^*^	−0.16; 6.60
Reduced working hours/shift	1.83	−0.72; 4.38			1.42^*^	−0.20; 3.04
Have to work overtime	0.34	−1.69; 2.38			1.17^*^	−0.10; 2.43
**Amount of income decreased due to COVID-19**	0.18	−0.47; 0.83	0.31	−0.31; 0.93	0.25	−0.15; 0.66
**IES-R score interpretation** (vs. Normal)						
Partial PTSD	4.36^***^	1.89; 6.83	3.31^***^	0.81; 5.82	3.68^***^	2.13; 5.23
Diagnosis of PTSD	8.47^***^	4.79; 12.15	6.92^***^	3.13; 10.70	8.06^***^	5.67; 10.46
Severe PTSD	10.88^***^	7.66; 14.09	12.50^***^	9.35; 15.65	9.15^***^	7.06; 11.25
**History of contact to COVID-19** (Yes vs. No)						
Contact with suspected COVID-19 infected people					2.07^**^	0.45; 3.69
Contact with objects which possibility contain the COVID-19 virus			5.91^***^	1.83; 9.98		

*** *p < 0.01*,

** *p < 0.05*,

* *p < 0.1*.

In the original article, there was an error.

A correction has been made to **3. Results, 3.1. Tables, Paragraph 03, line 131 and Paragraph 04, line 139**:

“Table 3 shows the mental well-being of respondents during COVID-19. Based on DASS 21 scale scoring, 4.9% of respondents were classified as having moderate to extremely severe levels of depression, 7.0% of respondents had moderate to extremely severe levels of anxiety, and 3.4% of respondents scored moderate to extremely severe levels of stress. Mean scores for depression, anxiety, and stress were 2.7 (SD = 6.5), 2.4 (SD = 6.3), and 4.7 (SD = 7.0), respectively”.

“Table 4 shows the factor associated with the depression, anxiety, and stress related to COVID-19. Married respondents had lower depression, anxiety, and stress levels than those that were single, separated, or widowed. Participants who are postgraduates had significantly higher stress levels than those with education level of high school and below (*p* < 0.05). A larger family size of more than 5 people was also linked to higher levels of depression, anxiety, and stress (*p* < 0.1) than those with family size of 1–2 people”.

The authors apologize for this error and state that this does not change the scientific conclusions of the article in any way. The original article has been updated.

